# Guidelines for Perioperative Care in Bariatric Surgery: Enhanced Recovery After Surgery (ERAS) Society Recommendations: A 2021 Update

**DOI:** 10.1007/s00268-021-06394-9

**Published:** 2022-01-04

**Authors:** Erik Stenberg, Luiz Fernando dos Reis Falcão, Mary O’Kane, Ronald Liem, Dimitri J. Pournaras, Paulina Salminen, Richard D. Urman, Anupama Wadhwa, Ulf O. Gustafsson, Anders Thorell

**Affiliations:** 1 Department of Surgery, Faculty of Medicine and Health, Örebro University, Örebro, Sweden; 2 Discipline of Anesthesia, Pain and Critical Care Medicine, Federal University of São Paulo, São Paulo, Brazil; 3 Dietetic Department, Leeds Teaching Hospitals NHS Trust, St James’s University Hospital, Leeds, UK; 4 Department of Surgery, Groene Hart Hospital, Gouda, Netherlands; 5 Dutch Obesity Clinic, The Hague, Netherlands; 6 Department of Upper GI and Bariatric/Metabolic Surgery, North Bristol NHS Trust, Southmead Hospital, Southmead Road, Bristol, UK; 7 Department of Surgery, University of Turku, Turku, Finland; 8 Division of Digestive Surgery and Urology, Turku University Hospital, Turku, Finland; 9 Department of Anesthesiology, Perioperative and Pain Medicine, Brigham and Women’s Hospital, Harvard Medical School, Boston, MA USA; 10 Department of Anesthesiology, Outcomes Research Institute, Cleveland Clinic, University of Texas Southwestern, Dallas, USA; 11 Division of Surgery, Department of Clinical Sciences, Danderyd Hospital, Karolinska Institutet, Stockholm, Sweden; 12 Department of Clinical Sciences, Danderyd Hospital, Karolinska Institutet, Stockholm, Sweden; 13 Department of Surgery, Ersta Hospital, Stockholm, Sweden

## Abstract

**Background:**

This is the second updated Enhanced Recovery After Surgery (ERAS®) Society guideline, presenting a consensus for optimal perioperative care in bariatric surgery and providing recommendations for each ERAS item within the ERAS® protocol.

**Methods:**

A principal literature search was performed utilizing the Pubmed, EMBASE, Cochrane databases and ClinicalTrials.gov through December 2020, with particular attention paid to meta-analyses, randomized controlled trials and large prospective cohort studies. Selected studies were examined, reviewed and graded according to the Grading of Recommendations, Assessment, Development and Evaluation (GRADE) system. After critical appraisal of these studies, the group of authors reached consensus regarding recommendations.

**Results:**

The quality of evidence for many ERAS interventions remains relatively low in a bariatric setting and evidence-based practices may need to be extrapolated from other surgeries.

**Conclusion:**

A comprehensive, updated evidence-based consensus was reached and is presented in this review by the ERAS® Society.

## Introduction

The use of bariatric surgery in the treatment for severe obesity has several benefits in terms of sustainable weight loss, improvements or resolution of several metabolic comorbidities as well as improved life expectancy [1, 2]. These benefits, in combination with continuously reducing complication rates, have led the way to a marked increase in the demand for bariatric surgical procedures worldwide [3].

The use of a multimodal stress-minimizing approach can reduce the rates of morbidity after major gastrointestinal surgery [4] and may shorten functional recovery as well as length-of-stay (LOS) in bariatric surgery [5, 6]. A first version of evidence-based guidelines for the perioperative care of patients undergoing bariatric surgery was published by the Enhanced Recovery After Surgery (ERAS) Society in 2016 [7]. Given the continued increase in bariatric surgery being performed worldwide, as well as the increasing popularity of novel surgical techniques, the evidence underpinning the recommendations is continuously evolving. Therefore, clinical guidelines need to be challenged and updated on a regular basis.

This document represents an updated consensus-based review of perioperative care for bariatric surgery based on best currently available evidence.

## Methods

Based on the first version of the ERAS Society guidelines for bariatric surgery published in 2016, the first and last author reviewed previous topics for a new update or removal and considered new topics for inclusion [7]. International authors with diverse expertise in the perioperative care of patients undergoing bariatric surgery (including surgery, anaesthesiology and nutrition) were invited by the ERAS Society to participate. All authors received instructions for the literature search and quality assessment [8].

### Search terms and sources

A principal literature search was performed utilizing the Pubmed, EMBASE, Cochrane databases and ClinicalTrials.gov through December 2020. Keywords included *“obesity”, “morbid obesity”, “bariatric surgery”, “metabolic surgery”, “gastric bypass”, “sleeve gastrectomy”, one anastomosis gastric bypass”, “mini gastric bypass”, “gastric banding”, “fast track”, and “enhanced recovery”.* Medical subheading terms were used as were accompanying entry terms for patient groups, interventions and outcomes. The references of each relevant study were also scrutinized for additional eligible studies. Meta-analyses of randomized controlled trials (RCTs) or observational studies, RCTs, and large cohort studies were eligible for inclusion. Retrospective, smaller cohort studies were considered when no higher-level evidence was available.

### Grading of evidence and recommendations

The quality of evidence supporting each recommendation was reviewed by one or two authors in conjunction with the first and last author. The Cochrane checklist was used to assess the methodological quality of each study [9]. The quality of evidence overall was then evaluated using the Grading of Recommendations, Assessment, Development and Evaluation (GRADE) system [10]. The level of evidence was categorized into four categories, high, moderate, low, or very low in accordance with the GRADE system [11].

The strength of the recommendations was likewise evaluated using the GRADE system. To generate strength of recommendations, all authors reviewed each recommendation with the accompanying evidence and GRADE rating of quality. The strength of each recommendation was determined by all authors and, if there was disagreement regarding the strength, a Delphi process was undertaken to reach consensus.

The criteria for rating strength of recommendations were as follows:

*Strong recommendation* The panel is confident that the desirable effects of adherence to the recommendation outweigh the undesirable effects.

*Weak recommendation*: The desirable effects to adherence to the recommendation probably outweigh the undesirable effects, but the panel is less confident.

## Results: evidence base and recommendations

The evidence and recommendations for ERAS items are presented in four different headings: preadmission, preoperative, intraoperative and postoperative and are numbered in the order they are to be used in clinical practice. Summary tables, Tables 1, 2, 3, 4, show an overview of the quality of evidence and grade of recommendation for each item.

**Table 1:** ERAS recommendations for preadmission care in bariatric surgery

Element	Recommendation	Level of evidence	Recommendation grade
1. Information, education and counselling	*Preoperative information and education, adapted to the individual requirements, should be given to all patients*	Low	Strong
2. Indications and contraindications for surgery	*Indications for bariatric surgery should follow updated global and national guidelines*	Moderate	Strong
3a. Smoking and alcohol cessation	*All patients should be screened for alcohol and tobacco use. Tobacco smoking should be stopped at least 4 weeks before surgery. For patients with alcohol abuse, abstinence should be strictly adhered to for 1–2 years. Moreover, the risk for relapse after bariatric surgery should be acknowledged*	Smoking: Moderate	Strong
		Alcohol: Low	Strong
3b. Preoperative weight loss	*Preoperative weight loss using very low or low-calorie diet prior to bariatric surgery should be recommended*	Postoperative complications: Moderate	Strong
	*While feasible, patients with diabetes and treatment with glucose-lowering drugs should closely monitor treatment effects, and be aware of the risk for hypoglycaemia. Very low calorie diet improves insulin sensitivity in patients with diabetes*	Postoperative weight loss: Low	Strong
		Diabetes: Low	Strong
4. Prehabilitation and exercise	*Although prehabilitation may improve general fitness and respiratory capacity, there is insufficient data to recommend prehabilitation before bariatric surgery*	Low	Weak

**Table 2:** ERAS recommendations for preoperative care in bariatric surgery

Element	Recommendation	Level of evidence	Recommendation grade
5. Supportive pharmacological intervention	*8 mg intravenous dexamethasone should be administered preferably 90 min prior to induction of anaesthesia for reduction of PONV as well as inflammatory response*	Glucocorticoids: Low	Weak
	*There is insufficient evidence to support perioperative statins for statin-naive patients in bariatric surgery. Patients on statins can safely continue the treatment during the perioperative phase*	Statins: Very low	Weak
	*Beta-adrenergic blockade does not influence the risk for adverse outcomes in bariatric surgery, but can be safely continued during the perioperative phase for patients at high risk of cardiovascular events*	Beta-adrenergic blockade: Low	Weak
6. Preoperative fasting	*Solids until 6 h before induction and clear liquids until 2 h before induction for elective bariatric surgery assuming no contraindications (e.g., gastroparesis, bowel obstruction)*	Low	Strong
	*Patients with diabetes should follow these recommendations, but further studies are needed for patients with additional risk factors such as gastroparesis*	Low	Strong
7. Carbohydrate loading	*There is insufficient evidence to make a recommendation about preoperative carbohydrate loading in bariatric surgery*	Low	Weak
8. PONV	*A multimodal approach to PONV prophylaxis should be adopted in all patients*	High	Strong

*PONV* Postoperative nausea and vomiting

**Table 3:** ERAS recommendations for intraoperative care in bariatric surgery

Element	Recommendation	Level of evidence	Recommendation grade
8. Perioperative fluid management	*The goal of perioperative fluid management is to maintain normovolemia and optimize tissue perfusion and oxygenation. Individual goal-directed fluid therapy is the most effective strategy, avoiding both restrictive or liberal strategies*	Moderate	Strong
	*Colloid fluids do not improve intra- and postoperative tissue oxygen tension compared with crystalloid fluids and do not reduce postoperative complications*	Low	Weak
9. Standardized anaesthetic protocol	*The current evidence does not allow recommendation of specific anaesthetic agents or techniques*	Low	Weak
	*Opioid-sparing anaesthesia using a multimodal approach, including local anaesthetics, should be used in order to improve postoperative recovery*	High	Strong
	*Whenever possible, regional anaesthetic techniques should be performed to reduce opioid requirements. Thoracic epidural analgesia should be considered in laparotomy*	Low	Weak
	*BIS monitoring of anaesthetic depth should be considered where ETAG monitoring is not employed*	Low	Strong
10 Airway management	*Anaesthetists should recognize and be prepared to handle the specific challenges in airways in patients with obesity*	Moderate	Strong
	*Endotracheal intubation remains the main technique for intraoperative airway management*	Moderate	Strong
11. Ventilation strategies	*Lung protective ventilation should be adopted for all patients undergoing elective bariatric surgery with avoidance of high PEEP values*	Moderate	Strong
	*Increases in driving pressure resulting from adjustments in PEEP should ideally be avoided*	Low	Strong
	*PCV or VCV can be used for patients with obesity with inverse respiratory ratio (1.5:1)*	Low	Strong
	*Positioning in a reverse Trendelenburg, flexed hips, reverse- or beach chair positioning, particularly in the presence of pneumoperitoneum, improves pulmonary mechanics and gas exchange*	Low	Weak
12. Neuromuscular blockade	*Deep neuromuscular blockade improves surgical performance*	Low	Strong
	*Ensuring full reversal of neuromuscular blockade improves patient recovery*	Moderate	Strong
	*Objective qualitative monitoring of neuromuscular blockade improves patient recovery*	Moderate	Strong
14. Surgical technique, volume and training	*Laparoscopic approach whenever possible*	High	Strong
	*During the learning curve phase, all operations should be supervised by a senior surgeon with significant experience in bariatric surgery*	Training: Low	Strong
	*There is a strong association between hospital volume and surgical outcomes at least up to a threshold value*	Hospital volume: Low	Strong
15. Abdominal drainage and nasogastric decompression	*Nasogastric tubes and abdominal drains should not be used routinely in bariatric surgery*	Weak	Strong

*PONV* Postoperative nausea and vomiting; *PEEP* Positive end-expiratory pressure; *PCV* pressure-controlled ventilation; *VCV* volume-controlled ventilation; *BIS* bispectral index; *ETAG* end-tidal anaesthetic gas

**Table 4:** ERAS recommendations for postoperative care in bariatric surgery

Element	Recommendation	Level of evidence	Recommendation grade
16. Postoperative oxygenation	*Patients without OSA or with uncomplicated OSA should be supplemented with oxygen prophylactically in a head-elevated or semi-sitting position. Both groups can be safely monitored in a surgical ward after the initial PACU stay. A low threshold for non-invasive positive pressure ventilation should be maintained in the presence of signs of respiratory distress*	Oxygen supplementation: Low	Strong
		Position in the postoperative period: High	
	*Patients with OSA on home CPAP therapy should use their equipment in the immediate postoperative period*	Moderate	Strong
	*Patients with obesity hypoventilation syndrome (OHS) are at higher risk of respiratory adverse events. Postoperative BiPAP/NIV should be considered liberally during the immediate postoperative period, in particular in the presence of hypoxemia*	Low	Strong
17. Thromboprophylaxis	*Thromboprophylaxis should involve mechanical and pharmacological measures. Doses and duration of treatment should be individualized*	High	Strong
18. Early postoperative nutritional care	*A clear liquid meal regimen can usually be initiated several hours after surgery*	Moderate	Strong
	*All patients should have access to a comprehensive nutrition and dietetic assessment with counselling on the macronutrient and micronutrient content of the diet based on the surgical procedure and the patient’s nutritional status*	Moderate	Strong
	*Patients and healthcare professionals should be aware of the risks of thiamine deficiency, especially in the early postoperative periods*	Low	Strong
19. Supplementation of vitamins and minerals	*A regimen of life-long vitamin and mineral supplementation and nutritional biochemical monitoring is necessary*	High	Strong
20a. PPI prophylaxis	*PPI prophylaxis should be considered for at least 30 days after Roux-en-Y gastric bypass surgery*	RYGB: Moderate	Strong
	*There is not enough evidence to provide a recommendation of PPI prophylaxis for sleeve gastrectomy, but given the high numbers of patients with gastroesophageal reflux after this procedure, it may be considered for at least 30 days after surgery*	SG: Very Low	Weak
20b. Gallstone prevention	*Ursodeoxycholic acid should be considered for 6 months after bariatric surgery for patients without gallstones at the time of surgery*	Moderate	Strong

*OSA* Obstructive sleep apnoea; *PACU* post-anaesthesia care unit; *CPAP* continuous positive airway pressure; *OHS* obesity hypoventilation syndrome; *BiPAP* bilevel positive airway pressure; *NIV* non-invasive ventilation; *LMWH* Low molecular weight heparin; *PPI* Proton pump inhibitor; *RYGB* Roux-en-Y gastric bypass; *SG* sleeve gastrectomy

### Preadmission items

#### Information, education and counselling

The patient scheduled for bariatric surgery must be well informed of the impact of extensive changes associated with life after bariatric surgery. They must be motivated and willing to participate in the long-term care, change in dietary patterns and to embrace the revised lifestyle after the operation. A preoperative educational program is often recommended in order to ensure realistic expectations, and to reduce anxiety, wound complications, postoperative pain and LOS [7, 12–16]. Results however, remain ambivalent. In a meta-analysis covering patients undergoing cancer surgery, education reduced anxiety and health care costs while simultaneously increasing the knowledge and satisfaction of patients [17]. A more recent RCT of 73 patients with colorectal cancer, reported improved body image after surgery and enhanced recovery of self-reported global health status and reduced LOS after extra preoperative group education [18]. However, meta-analyses on education for patients undergoing elective spinal surgery as well as a cluster RCT in patients undergoing visceral surgery did not report any sustained benefit on the majority of clinical outcome [19, 20]. The educational intervention proves hard to standardize, and there is a paucity in research assessing its benefits for bariatric surgery.

While the grade of evidence remains low, a formal psycho-social evaluation including environmental, familiar, nutritional and behavioural factors in line with current recommendations from the American Society for Metabolic and Bariatric Surgery (ASMBS) should be performed prior to bariatric surgery [21].

Despite the low grade of evidence, preoperative information and education are strongly recommended as a necessary step of informed consent in order to improve knowledge, ensure adequate risk perception, and allow active patient participation in making well-informed choices (Table 1).

#### Indications and contraindications for surgery

The current indications for bariatric surgery are BMI ≥ 40 kg/m^2^ alone or BMI ≥ 35 kg/m^2^ and an obesity-related comorbidity that could be expected to improve/resolve by surgery-induced weight loss maintenance. These were established in a consensus meeting in 1991 and subsequently confirmed by more recent guidelines [22, 23]. The major addition to the indications for bariatric or rather, metabolic surgery, has been provided by the international consensus conference of Diabetes Surgery Summit, established in 2007 recommending the use of gastrointestinal surgery to treat type-2 diabetes mellitus (T2D), including individuals with class-I obesity. The joint statement by international diabetes organizations in 2016 recommends that metabolic surgery should be considered as an option to treat T2D in patients with class-I obesity (BMI 30.0–34.9 kg/m^2^) and inadequately controlled hyperglycaemia despite optimal medical treatment [24] (Table 1).

#### Preoperative optimization

##### Smoking and alcohol cessation

Cessation of smoking at least 4–8 weeks before surgery reduces postoperative complications after non-bariatric surgery, in particular wound and cardiovascular complications [25]. For bariatric surgery, smoking has been associated with increased risk of marginal ulcers, infectious and respiratory complications [26, 27]. Although there is a lack of RCTs addressing smoking cessation prior to bariatric surgery, a recent systematic review of 28 non-RCTs [28] reported a reduction in postoperative morbidity. While the optimal timing of cessation remains unknown, an intervention beginning at least 4 weeks before surgery including weekly counselling and use of nicotine replacement therapy is the most likely approach to impact complications and long-term smoking cessation [29]. Despite best efforts, achieving cessation appears to be difficult, in particular in the long term [30] (Table 1).

High alcohol consumption can increase the risk of postoperative complications—mainly infectious and related to wound healing [31]. The effect of preoperative alcohol cessation was addressed in a Cochrane review including three RCTs with a combined total of 140 patients who underwent colorectal or orthopaedic surgery. The pooled estimate from these studies suggested a significant reduction in risk (RR 0.62, 95%CI 0.40–0.96) for postoperative complications following preoperative counselling and a short, but intensive intervention for patients with increased consumption [32]. The complex nature of mandatory behavioural changes in combination with an increased risk of postoperative alcohol overconsumption and dependency in particular for patients with previous substance abuse, is the basis for the current recommendations of a 1–2 year period of documented alcohol abstinence for patients with earlier overconsumption [33]. The level of evidence for this recommendation remains low (Table 1).

##### Preoperative weight loss

In contrast to so-called Insurance-mandated preoperative weight loss programs, a 2–4 week period of Low Calorie Diet (LCD, 1000–1200 kcal/d) or Very Low Calorie Diet (VLCD, 800 kcal/d) is usually recommended prior to bariatric surgery. This regimen has been shown to reduce liver volume and a surgeon´s perceived complexity of the procedure [34, 35]. Furthermore, VLCD for two weeks is associated with improved whole-body insulin sensitivity as demonstrated in another RCT [36]. In a previous systematic review which included 11 non-randomized patient cohorts, preoperative weight loss was associated with reduced postoperative complications [37], which has later been confirmed in one RCT and two large retrospective studies [38–40]. The effect may be more pronounced in patients with a higher BMI [40].

However, two recent meta-analyses and one retrospective study failed to demonstrate the effect of diet on postoperative morbidity [41, 42], but one of the meta-analyses reported a statistically significant (27%) reduction in LOS in patients submitted to preoperative weight loss [43].

An improvement in postoperative weight loss in patients who lost weight preoperatively has been reported from observational studies [39, 44]. The effect of preoperative weight loss was also evaluated in an ERAS setting showing reduced operating time as well as improved postoperative weight loss [41]. There also seems to be an improvement in postoperative weight loss in patients who achieve preoperative weight loss > 10% [39, 44].

The effect of preoperative weight loss in patients with obesity and T2D has not been specifically addressed in any RCT or large retrospective study.

The optimal composition of preoperative low-calorie diet is yet to be determined. Most protocols adhere to a commercially available composition, but data from comparisons between these are sparse [45].

Overall, there is high level of evidence that 2–4 weeks of either a LCD or a VLCD reduces liver volume, moderate evidence of a reduction of postoperative complications, and low-quality evidence of postoperative weight loss (Table 1).

### Prehabilitation and exercise

The concept of prehabilitation includes interventions aiming at increasing physical functioning before surgery, which in turn may improve recovery and reduce morbidity postoperatively. In a recent meta-analysis, including all RCTs in patients undergoing various types of abdominal surgery published between 1966 and 2017, a reduction in overall postoperative morbidity in the prehabilitation group (OR 0.63, 95%CI 0.46–0.87) with a composite pulmonary morbidity endpoint (OR 0.40, 95%CI 0.23–0.68), and borderline significance for reduction in LOS was seen [46]. There was a heterogeneity between protocols with a length of the preoperative interventions between 2 and 4 weeks. No difference was found for the six-minute walking test. However, a previous meta-analysis indicated that complications as well as LOS were reduced in patients undergoing prehabilitation [47].

Some relatively small RCTs have addressed the effect of prehabilitation in patients scheduled for bariatric surgery. A 12-week exercise program including endurance training was associated with reduced weight, cardiometabolic risk factors as well as improved general physical fitness [48, 49]. A 6-week preoperative training was also associated with maintained improvement in physical activity 6 months postoperatively [50]. Specific inspiratory muscular training preoperatively improved immediate (12 h) postoperative oxygenation and increased inspiratory muscular strength [50]. None of these studies have reported outcome in terms of recovery or morbidity.

Although prehabilitation is a promising intervention which seems to have the potential to reduce morbidity in some surgical settings, the extrapolation of the results to patients undergoing bariatric surgery remains questionable (Table 1).

### Preoperative items

#### Supportive pharmacological intervention

In order to reduce the stress response during and after surgery, several pharmacological interventions have been suggested, as described below.

##### Glucocorticoids

Glucocorticoids are known for their anti-inflammatory properties, thus potentially reducing the perioperative stress response. In patients undergoing surgery for gastrointestinal cancer, corticosteroids administered preoperatively or following induction of anaesthesia has been associated with fewer complications and a milder systemic inflammatory response (SIR) [51, 52]. In elective surgery for inflammatory bowel disease, a single dose of 8 mg dexamethasone upon induction of anaesthesia reduced postoperative ileus, the intensity of postoperative pain, and LOS [53]. Perioperative dexamethasone for total joint arthroplasty patients was also associated with reduced LOS, postoperative pain and stress response as reported in one meta-analysis including 17 RCTs [54]. A single dose of dexamethasone seems safe as the side effects were limited to a physiological rise in blood glucose levels [54]. Furthermore, previous systematic reviews and meta-analyses have demonstrated that administration of dexamethasone preoperatively did not increase postoperative (wound or systemic) infections or anastomotic leakage [55–57]. There is still a paucity of studies reporting the perioperative use of dexamethasone in bariatric surgery.

##### Statins

In a systematic review, perioperative use of statins in patients undergoing various types of abdominal surgery was associated with reduced mortality, systemic infection and anastomotic leak [58]. Subsequent cohort studies further confirmed the association between perioperative statin usage and a reduction of postoperative complications [59–64]. However, two RCTs comparing statins vs placebo in patients undergoing non-cardiac surgery and major colorectal surgery found no difference in postoperative complications [65, 66], although simvastatin attenuated the early proinflammatory response to surgery after colorectal surgery [65]. Furthermore, a meta-analysis of 5 RCTs found no evidence for statins to reduce the postoperative risk of infection [67]. Literature on statin use in bariatric surgery specifically is absent. Routine perioperative administration of statins to patients undergoing bariatric surgery for prevention of complications is therefore not recommended.

##### Beta-blockade

It has been hypothesized that beta-blockers, by decreasing the effect of surgical stress on the heart, can reduce complications such as myocardial infarction, stroke and cardiac arrhythmias. The most recent meta-analysis of 83 RCTs, including an international multicentre trial in which a potential harmful effect (increased mortality and risk of stroke) was detected for preoperative use of metoprolol, also reported low-certainty evidence for a reduction in atrial fibrillation and myocardial infarction after beta-blocker usage in non-cardiac surgery [68, 69]. Bradycardia and hypotension were both increased with low- and moderate-certainty level evidence, respectively. The evidence for early all-cause mortality was uncertain. Subsequent cohort studies assessing the continuous use of beta-blockers in gastrointestinal cancer surgery reported decreased risk of postoperative cardiovascular complications, anastomotic leakage and sepsis and reduced mortality rates at 90-days and 1-year after surgery [70, 71]. Only one retrospective study assessed the association between preoperative beta-blocker therapy and postoperative outcome for patients undergoing laparoscopic RYGB. There was no evidence for a beneficial effect on moderate- and long-term survival and postoperative complications [72]. At present, it is not recommended to routinely use perioperative beta-blockers in patients undergoing bariatric surgery. However, patients at high risk of cardiovascular events who are already on beta-blockade can safely continue this treatment through the perioperative process [73].

#### Preoperative fasting

Previous studies have demonstrated no differences in residual gastric fluid volume (RGFV), pH [74, 75] or gastric emptying rates following an intake of semi-solid meals [76, 77] or drinks [78] in patients with obesity when compared to patients with normal weight. No differences were found in RGFV and pH in a RCT of patients with severe obesity who drank 300 ml of clear fluid 2 h before induction of anaesthesia, compared with those who fasted after midnight [79, 80]. RGFV and pH were also similar following an overnight fast in patients with obesity and diabetes (with and without autonomic neuropathy) compared with controls without diabetes [80–82].

While the level of evidence remains low, preoperative fasting for solids (equivalent to a light meal) of at least 6 h and clear fluids of 2 h before induction of anaesthesia is recommended, if there are no contraindications (Table 2).

#### Carbohydrate loading

Preoperative carbohydrate conditioning, using iso-osmolar drinks (CHO) ingested 2–3 h before induction of anaesthesia, attenuated development of postoperative insulin resistance, reduced postoperative nitrogen and protein losses and maintained lean body mass [83]. Two meta-analyses demonstrated CHO to be associated with reduction in hospital LOS by about 1 day after major abdominal surgery [84, 85]. A similar reduction was reported from a small RCT of 20 patients undergoing SG [86]. When CHO were administered to patients with T2D (mean BMI 28.6 kg/m2), no differences were noted in gastric emptying times compared with healthy subjects [87]. However, postprandial glucose concentrations reached a higher peak and were elevated for longer time in patients with diabetes [87]. In addition, CHO did not lead to an increase in aspiration-related complications in patients undergoing laparoscopic RYGB, even in patients with diabetes and delayed gastric emptying [88–90]. Two further studies have used CHO in bariatric surgery within an enhanced recovery pathway [91, 92]. In a RCT comparing enhanced recovery versus standard care in bariatric patients (including CHO), no differences in overall complication rate were noted [91]. In addition, compliance with CHO was only 15% [91]. Another study of 90 patients randomized to either carbohydrate-rich drink, protein-rich drink or tap water did not show any effect on nausea after gastric bypass surgery [93].

Currently, there is insufficient evidence to support routine use of preoperative carbohydrate loading in bariatric surgery (Table 2).

#### Prevention of nausea and vomiting

Patients undergoing bariatric surgery are frequently female and non-smokers, undergoing laparoscopic or robotic procedures of more than one hour in duration and receive perioperative opioid analgesia—all of which are risk factors for PONV. Gastric surgery, history of acid reflux and reduction in gastric size, in particular after sleeve gastrectomy may further contribute to PONV [94–96].

Recent guidelines recommend a multimodal approach including total intravenous anaesthesia with Propofol (TIVA), avoidance of volatile anaesthetics and fluid overload, and minimization of intra- and postoperative opioids [97].

Compared to volatile opioid anaesthesia with opioids, opioid-free TIVA was associated with a significantly lower rate and severity of PONV in a RCT including 119 patients [98]. In addition, one antiemetic agent from three of the following six classes is recommended: 5-hydroxytryptamine receptor antagonists, long-acting corticosteroid like dexamethasone, butyrophenones, neurokinin-1 receptor antagonists, antihistamines and anticholinergics [99]. In addition, multimodal analgesia and regional anaesthesia techniques are recommended, as opioid-sparing strategies to further reduce the risk of PONV [99].

The evidence for a multimodal PONV regimen based on current RCTs is strong, but there are limited data on the use of TIVA (Table 2).

### Intraoperative items

#### Perioperative fluid management

Obesity can cause changes in different fluid compartments and affect body composition, leading to an increase in absolute fluid volume and subsequently in cardiac output [100]. Therefore, it remains a challenge in the perioperative period to estimate fluid requirements needed to maintain normovolemia and hence optimized tissue perfusion and oxygenation.

Intraoperative hyper- as well as hypovolemia is associated with worse outcome [101–103]. There is some evidence to suggest that restrictive fluid administration in both non-bariatric [104] and bariatric surgery [105] can increase complications as well as LOS and mortality. On the other hand, high intravenous volume of fluids administered on the day of surgery is associated with increased LOS as well [106].

At present, individualized goal-directed fluid therapy (GDFT) is the most effective way to optimize cardiac performance and to improve oxygen delivery in the perioperative period [107].

In a RCT of 60 patients undergoing laparoscopic bariatric surgery, GDFT was associated with improved tissue oxygenation in the early postoperative period [108]*.* Additionally, GDFT guided by stroke volume optimization according to arterial pressure waveform analysis or by Pleth Variability Index (PVI) can decrease the incidence of postoperative nausea and vomiting and shorten hospital LOS [109]. GDFR can continue in the surgical ward guided by non-invasive measurements [21].

Regarding the type of fluid, crystalloids leave the circulation more quickly than colloids, and therefore, may increase the risk of tissue oedema and impaired tissue oxygenation [110]. The intravascular effects of colloids are context-sensitive and therefore affected by fluid status [111]. During hypovolemia, colloids remain longer intravascularly [112] and might consequently better maintain hemodynamic stability, which might in turn lead to improved tissue perfusion and oxygenation [113, 114]. There is a paucity of studies comparing crystalloid and colloid solution in bariatric surgery. No difference in intra- and postoperative subcutaneous tissue oxygen tension (PsqO_2_) [107], cytokine and inflammatory marker levels [115], or postoperative complications [116] has been seen when comparing crystalloids with colloids or hydroxyethyl starch during abdominal surgery. Data from several RCTs suggest the benefit of using balanced crystalloids and limiting the use of 0.9% normal saline [117–119].

There is moderate evidence supporting an individual goal-directed fluid therapy, avoiding both restrictive and liberal strategies (Table 3).

#### Standardized anaesthetic protocol

Short-acting agents and minimal opioid use during the operation is of importance to enhance recovery. Anaesthesia induction should preferably be based on lean body weight to avoid hypotension [120], while using TBW may be more appropriate for a maintenance infusion [121]. Classic target-controlled infusion (TCI) models have poor predictive ability when used in patients with obesity [122]. Propofol is the most frequently used induction agent, and it has not be shown to increase the incidence of propofol infusion syndrome-related rhabdomyolysis in patients with severe obesity during standard bariatric surgery [123]. If volatile anaesthetics are used for maintenance, desflurane may offer faster wake up times compared to sevoflurane or isoflurane in patients with a BMI > 30 kg/m^2^ [124]. However, unlike sevoflurane with its bronchodilator effects, desflurane can induce increased airway resistance as well as hypertension and tachycardia. The decision regarding which inhalational agent to use should therefore be determined based on existing comorbidities and other related factors.

Bispectral index (BIS) represents one of several ways to monitor depth of anaesthesia, with the goal of decreasing intraoperative awareness and reducing the amount of administered anaesthetic [125]. For monitoring of intraoperative awareness, BIS or end-tidal anaesthetic gas (ETAG) monitoring might be used. Both of these have been shown to similarly reduce rates of intraoperative awareness compared to using only clinical signs [125, 126].

Patients with obesity generally show an increased sensitivity towards opioid sedative effects and consequently higher susceptibility towards respiratory depression [127]. In an effort to reduce the incidence and severity of postsurgical pain, multimodal analgesia using limited doses of opioids has been advocated [128–130]. Lidocaine, dexmedetomidine, ketamine and magnesium, when used as part of opioid-free anaesthesia, may have better anti-inflammatory effects than classical opioid-based anaesthesia and may therefore be preferable [131]. In addition, appropriate use of nonsteroidal anti-inflammatory drugs (NSAIDs) reduces opioid consumption [132, 133]. One limitation of most NSAIDs is that they are “low-ceiling” analgesics. Paracetamol is free of bleeding, gastric and renal side effects that limit the use of NSAIDs.

Regional anaesthetic techniques have been demonstrated to be highly efficient in reducing opioid requirements. Epidural analgesia for postoperative pain is effective but is not required in laparoscopic surgery. Ultrasound-guided transversus abdominis plane block can decrease pain scores and opioid requirement, and improve ambulation after bariatric surgery [134]. Infiltration of bupivacaine 0.5% before incision results in a reduction in opioid consumption and postoperative pain [135]. Other promising strategies are intraperitoneal instillation of bupivacaine [136] and erector spinae plane block [137].

Although current evidence does not allow recommending of specific anaesthetic agents or techniques, there is high level of evidence in support of using multimodal, opioid-sparing analgesia approaches to improve postoperative recovery (Table 3).

#### Airway management

Some studies have reported an association between severe obesity and difficult intubation [138]. In a comprehensive analysis of a single centre experience, the overall incidence of difficult intubation in patients with severe obesity was 4.2% and difficult mask ventilation 2.9% [139]. Factors associated with difficult intubation were age > 46 years, male gender, airway class 3–4 according to the Mallampatti score, thyromental distance (distance from the thyroid notch to the tip of the jaw with the head extended) < 6 cm and presence of intact dentition. Male patients with severe obesity, especially ones with BMI more than 50 and OSA, as well patients with a neck circumference > 42 cm had a higher risk of difficult mask ventilation and intubation [139]. The use of simple nasal or high flow nasal cannula should be considered as an adjunct during mask ventilation in patients with a suspected or known difficult intubation. This has been demonstrated to help maintain oxygenation by increasing apnoea time up to 40% and can reduce peri-intubation desaturation during anaesthesia induction [140, 141].

The use of a videolaryngoscope (VL) may improve glottis visualization of the trachea and increase first attempt success rate of intubation compared to regular laryngoscope blades, especially in the setting of a difficult airway [142, 143]. However, regarding its efficacy results remain heterogeneous [144]. If a tracheostomy needs to be performed in a patient with significant obesity, it may take much longer than it would in a patient without obesity and it is associated with higher complication rates [145].

If baseline oxygen levels cannot be maintained immediately after extubation, use of continuous positive airway pressure (CPAP) therapy is recommended. Positive airway pressure should be continued until the patient’s respiratory rate and effort return to normal and there are no episodes of hypopnea and apnoea for at least one hour [146] (Table 3).

#### Ventilation strategies

While different strategies may play a role for protective ventilation, suggested measures to protect lungs from ventilated-induced lung injury include low tidal volumes (V_T_) and low positive end-expiratory pressure (PEEP) level without recruitment manoeuvres [147, 148].

Lung volume does not increase proportionally with body weight in patients with obesity [149]. Using the Predicted Body Weight (PBW), which takes in to consideration the patient´s height and sex, rather than the actual body weight, may be preferred when estimating V_T._ A low physiological V_T_ can be lung protective in patients with acute respiratory distress syndrome (ARDS) [150], as well as in patients with healthy lungs under general anaesthesia [151, 152]. Current evidence suggests that a tidal volume in the range of 6–8 mg/kg of PBW can reduce pulmonary complications and should be employed for all patients with healthy lungs regardless of obesity [153].

Patients with obesity are predisposed to developing atelectasis mainly in dependent lung regions, making the combination of recruitment manoeuvers (RMs) and PEEP a strategy to improve gas exchange and lung mechanics [154–156]. There is much uncertainty regarding the optimal level of PEEP for patients with obesity and healthy lungs, and the role of PEEP and RM to avoid postoperative pulmonary complications remains unclear [157]. The PEEP requirements vary extensively among patients [158]. Receiving low V_T_ during anaesthesia and individualized PEEP settings can reduce postoperative atelectasis while improving intraoperative gas exchange and driving pressures, suggesting that individualized levels of PEEP targeted to physiological goals could be used to protect the lungs [158].

High driving pressure (the difference between plateau pressure and PEEP) may be associated with an increased risk of severe adverse outcomes in patients with acute respiratory distress syndrome [159] as well as in patients undergoing elective surgery [152, 160]. Patients with obesity may require higher cut-off values of protective driving pressure than patients without obesity due to low lung capacity or physiologic changes occurring during the surgical procedure [161].

Pressure-controlled ventilation (PCV) may promote more homogeneous ventilation within different lung compartments, which in turn mitigates alveolar overdistention and improves oxygenation [162, 163]. On the other hand, volume-controlled ventilation (VCV) allows better control of V_T_ during procedures intermittently affecting chest wall compliance and might be associated with lower incidence of postoperative pulmonary complications [164].

There is moderate evidence in support of using lung protective ventilation with avoidance of high values of PEEP. PCV or VCV can be used with inversed respiratory ratio, ideally avoiding increases in driving pressure from adjustments in PEEP, but the level of evidence remains low (Table 3).

#### Neuromuscular blockade

Neuromuscular blockade (NMB) is essential for laparoscopic or robot-assisted surgery for weight reduction [165, 166]. Even though controversial studies exist, current data are suggestive of benefit with deep NMB in patients undergoing bariatric procedures [167–172].

Deep NMB requires its prompt and complete reversal at the end of surgery. While the effects of residual NMB have not been specifically studied in bariatric surgery, many of the physiological findings associated with this condition may have increased relevance to the bariatric surgery population [173–175]. This puts patients with severe obesity at an increased risk of postoperative pulmonary complications, such as pulmonary atelectasis, pneumonia and even respiratory failure [176]. Patients should be fully reversed and carefully monitored with objective methods of residual neuromuscular blockade assessment during surgery and following reversal at the end of surgery [7, 168].

Nerve-stimulated TOF-ratio ≥ 0.9 translates into recovery benefits by avoiding recurarization and reintubations related to persistent blockade [177–180]. Sugammadex reverses moderate block 6.5 times faster than neostigmine, and deep neuromuscular blockade 16.8 times more rapidly than neostigmine [181], and have been associated with fewer adverse events compared with traditional reversal agents [181–183].

Sugammadex dose should be adjusted to the level of NMB and body weight to allow complete and rapid reversal. A dose of 2 mg/kg IBW + 40% seems to provide balance between speedy and complete recovery and favourable side effect profile [184–186]. The incidence of confirmed hypersensitivity is around 5% and anaphylaxis 0.3%, with the anaphylaxis occurring only with the dose of 16 mg/kg [187, 188]. In reality, the incidence of hypersensitivity reactions seems to be reported at a much lower rate and a dose of 2 mg/kg IBW + 40% seems to be most appropriate for the bariatric surgery population.

Deep NMB should generally be employed, with the understanding that it may not be reversible with traditional reversal agents until TOF is ≥ 3, while reversal with sugammadex would allow faster recovery and optimal operating room time (Table 3).

#### Surgical technique, volume and training

Laparoscopic surgery is today the self-evident gold standard in bariatric surgery. However, most studies comparing laparoscopic and open technique in bariatric surgery were performed in the beginning of the laparoscopic era. Thus, the current evidence level regarding the comparison between laparoscopic and open surgery hardly corresponds to current clinical practice. Compared to open surgery, there is moderate-quality evidence that laparoscopic approach in bariatric surgery is associated with a shorter LOS and earlier recovery, and high-quality evidence of an association with reduced rate of wound infections and hernias. For complications in general, duration of surgery and reoperation risk, the limited evidence is in favour for laparoscopy [189–192].

In 2016, the three most commonly performed primary surgical bariatric/metabolic procedures worldwide were sleeve gastrectomy (SG, 54%), Roux-en-Y gastric bypass (RYGB, 30%) with 30%, and one anastomosis gastric bypass (OAGB, 5%), respectively [193].

There are no studies comparing the feasibility of enhanced recovery between different bariatric procedures. The two most recent meta-analyses identified five RCTs and 12 observational studies assessed the application of ERAS for patients undergoing bariatric surgery and almost all of these studies included patients undergoing SG or RYGB [5, 194]. The safety and benefits of ERAS protocols in OAGB were assessed in one prospective study reporting shorter LOS and reduced emergency room visits and readmissions after surgery [195], and in one comparative study between OAGB and SG showing that the program was equally safe with both procedures [196]. The benefits and safety with the use of an ERAS protocol have been shown after all of the most common bariatric surgeries [5, 194].

There are limited data on the effect of hospital volume on perioperative safety in an ERAS environment [197]. However, the effect of hospital volume in traditional perioperative care has been actively studied for bariatric surgery. A recent study evaluated almost 40,000 bariatric surgery procedures performed in 19 high-volume centres, reported bench mark complication rates of 7.2% for RYGB and 6.2% for SG [198]. A systematic review [199] showed evidence of improved patient outcomes in high-volume surgeons and institutions, which was also confirmed by a large nationwide registry study [200]. Thus, there is low quality evidence in support of improved outcome at high-volume centres.

While recognizing a fast track pathway, many centres are involved in the training of new bariatric surgeons. During the learning curve process, longer operation times and even higher complication rates might be expected [201]. Previous experience with laparoscopic surgery, as well as adopting an individualized and comprehensive training programme, may improve surgical technical skills [202, 203]. Furthermore, active coaching and mentoring from experienced bariatric surgeons may result in shorter operative time and lower complication rates during surgical training [204–207] (Table 3).

#### Abdominal drainage and nasogastric decompression

The sensitivity of abdominal drainage (between 0 and 94%) in detecting postoperative leakage after RYGB has previously been assessed in a systematic review including 18 cohort studies [208]. A subsequent observational study including more than 140.000 patients showed no beneficial effects of routine abdominal drainage after bariatric surgery, but rather an increased morbidity rate [209]. Two small RCTs comparing routine use of abdominal drains to no drains reported similar complication rates, but more postoperative pain for the groups with drains [210, 211]. One cohort study [212] and one RCT [213] could not confirm any reduction in anastomotic leak with nasogastric decompression in patients undergoing bariatric surgery. There is no evidence supporting routine abdominal drainage or nasogastric decompression following bariatric surgery (Table 3).

### Postoperative items

#### Postoperative oxygenation

Obesity is associated with increased work of breathing as well as higher risk of perioperative atelectasis persisting for longer duration compared to patients with normal weight [214, 215]. In addition, OSA is a common condition among patients with severe obesity [216]. It is associated with increased risk of cardiopulmonary events and a significant mortality rate, in particular in cases with high Apnoea-Hypopnea index (AHI) [217]. The STOP-BANG (Snoring, Tiredness during daytime, Observed apnoea, high blood Pressure, Body mass index, Age, Neck circumference, Gender) questionnaire might be used in the preoperative evaluation to identify patients with high risk of suffering from this comorbidity [218]. Patients with OSA have historically been considered to be at a high risk of perioperative complications, particularly those of respiratory nature [7]. Patients with obesity hypoventilation syndrome may exhibit even higher risk of cardiopulmonary complications and longer hospital stay, compared to patients with OSA alone [219].

Modern minimally invasive surgical techniques, combined with an emphasis on opioid-sparing analgesic approaches, and the use of CPAP/BiPAP treatment when necessary, can decrease the risk of cardiopulmonary complications in patients with OSA who undergo bariatric surgery [220].

Since the majority of the potentially dangerous hypoxic events occur in close proximity to the discontinuation of anaesthesia or after opioids are given, a standard or slightly prolonged observation in the PACU will be sufficient for most patients [221]. A postoperative positioning in a head-elevated, semi-seated position prevents further development of atelectasis and may improve oxygenation [215]. Supplemental oxygen improves oxygen saturation but may increase the duration and time to detection of apnoea/hypopnoea as well as carbon dioxide retention and should therefore be used with caution [222]. Positive airway pressure treatment can be used to prevent hypoxic events in the postoperative phase and should be continued in patients using CPAP/BiPAP treatment before surgery in order to reduce the risk for apnoea and other complications [223–225].

In addition, in patients with hypoxemia (defined as an oxygen saturation < 90%) during the immediate postoperative period, non-invasive positive pressure treatment such as CPAP or NIPPV (with or without supplemental oxygen) should be used liberally [226]. Standardized discharge criteria can be used to determine when the patient is ready to be discharged from the PACU, in addition to a satisfactory clinical evaluation to ensure that the patient has stable vital signs, including adequate respiratory rate and depth. Finally, as stated previously, it is recommended to minimize systemic opioid use in order to reduce episodes of apnoea/hypopnea (Table 4).

#### Thromboprophylaxis

Thromboembolic complications continue to represent a main cause of morbidity and mortality after bariatric surgery [227]. Risk factors, in addition to obesity itself, include history of venous thromboembolism, increased age, smoking, varicose veins, heart or respiratory failure, OSA, thrombophilia and oestrogen oral contraception [228].

There is wide variation in bariatric surgery practice, particularly in terms of treatment duration and dose adjustment [229, 230], and there is paucity of the literature for high-quality studies to inform clinical practice [231].

A Cochrane review from 2018 concluded with moderate-quality evidence that combining intermittent pneumatic leg compression and pharmacological prophylaxis decreases the incidence of deep venous thrombosis (DVT), and pulmonary embolism [232]. In addition, a different Cochrane review concluded that with high-quality evidence graduated compression stockings by itself are effective in reducing the risk of DVT in hospitalized patients who have undergone general surgery [233].

The ASMBS guideline suggests thromboprophylaxis, including unfractionated heparin or low-molecular-weight heparin (LMWH) given within 24 h postoperatively, for all patients after bariatric surgery [21].

A systematic review including 20 studies suggested that for thromboprophylaxis with LMWH, enoxaparin 40 mg twice daily, dalteparin 5000 IE twice daily or tinzaparin 75 IU/kg once daily should be considered for patients with BMI ≥ 40 kg/m^2^ [234].

A study of 105 patients using antifactor Xa (aFXa) assay demonstrated that BMI-based thromboprophylactic dosing of enoxaparin after bariatric surgery could be suboptimal in 15% of patients and overdosing was more common than underdosing [235]. For optimization of dosage, it has been suggested that in high-risk bariatric surgery patients, the measurement of aFXa should be considered [235, 236].

In a large study, it was reported a 28-fold increase in mortality risk in patients with venous thromboembolic events and that more than 80% occurred after discharge [237]. Therefore, routine post-discharge pharmacoprophylaxis extending beyond standard treatment should be considered for high-risk patients [21, 237]. This is also supported from mechanistic data and studies of surgical patients undergoing non-bariatric surgery [238–240].

Regarding the use of retrievable inferior vena cava filters in the context of bariatric surgery, a systematic review suggested that there was no evidence to suggest that the potential benefits outweigh the significant risks [241].

An emerging thromboembolic complication is portomesentric and splenic vein thrombosis. A systematic review suggested that it is most common after SG and that the portal vein is the most commonly involved vessel [242]. Another systematic review focusing on SG exclusively suggested that the incidence ranged from 0.37% to 1% [243]. Further studies on the impact of prophylaxis strategies to reduce this specific complication are needed (Table 4).

#### Early postoperative nutritional care

As part of the assessment and preparation for bariatric surgery, patients should have access to a comprehensive nutrition and dietetic assessment [244–249]. A clear liquid meal regimen can usually be initiated a couple of hours postoperatively before moving on to nourishing fluids [245, 250]. The dietetic consultations will include advice on texture progression specific to the surgical procedure and the bariatric centre’s usual practice [245, 250].

Patients will continue to progress the introduction of food and different textures at home. They are encouraged to eat slowly, chew their food well and avoid drinking with food [245, 250]. In the early postoperative weeks, patients are at risk of developing thiamine deficiency because of the relatively small depots in combination with fast weight loss and poor nutritional intake. This risk is further increased in the presence of vomiting, diarrhoea or non-adherence to the vitamin and mineral supplements [244–247, 250]. If risk of thiamine deficiency is suspected, it must be treated immediately.

The dietitian will advise on protein intake. Generally, following the adjustable gastric band, SG and RYGB, at least 60–80 g/day total protein intake or 1.0–1.5 g/kg ideal body weight (IBW) is recommended. However, hypoabsorptive procedures such as the biliopancreatic diversion with duodenal switch, OAGB and single anastomosis duodenal-ileal bypass increase the risk of protein-energy malnutrition [251, 252]. Consequently, a protein intake of at least 90 g/day or as high as 2.1 g/kg IBW is then recommended [245] (Table 4).

#### Supplementation of vitamins and minerals

Postoperative dietetic follow-up is essential. After bariatric surgery, there is an increased risk of deficiencies of iron, folate, vitamin B12, vitamin D and trace minerals zinc, copper and selenium. Hypoabsorptive procedures can further increase the risk of vitamin A, E and K deficiencies [251, 252]. Consequently, patients are required to adhere to a regimen of life-long vitamin and mineral supplementation and nutritional biochemical monitoring. The supplements and biochemical monitoring differ by surgical procedure and full details may be found in bariatric surgery nutritional guidelines [244, 247] (Table 4).

#### Postoperative prophylaxis

##### Proton pump inhibitors

A systematic review reported an overall incidence rate of marginal ulcers of 4.6% after RYGB. However, the range between included studies varied from 0.6–25% [253]. Several studies have reported a significant reduction in marginal ulcers if PPIs are used prophylactic in the perioperative phase in particular when used for longer duration such as 3 months [254, 255]. However, when using a standardized surgical technique, with a small gastric pouch, the need for PPI prophylaxis has been questioned [256]. While recognizing the weak evidence of support, prophylactic use of PPI is safe and without significant cost. These medications can therefore be considered for postoperative prophylaxis after RYGB. If used, higher doses than standard should be given after gastric bypass surgery due to the reduced uptake [257]. In addition, opening of the capsules could improve postoperative uptake and should be considered [258].

There are no studies addressing the benefits of PPI use following sleeve gastrectomy. While high rates of reflux and reflux-related complications are reported in some studies [259–262], there is insufficient evidence to give any firm recommendations on the use of PPI after sleeve gastrectomy (Table 4).

##### Gallstone prevention

Five RCTs (four addressing RYGB, and one SG) including a total of 616 patients reported significant reduction in postoperative gallstone formation by the use of ursodeoxycholic acid in patients without gallstones at the time of surgery [263–267]. While the optimal dose remains controversial, these studies suggest that 500–600 mg may be sufficient. The results are further strengthened by a meta-analysis addressing three studies for RYGB and three for SG of different study designs showing a benefit for patients prescribed ursodeoxycholic acid for postoperative prophylaxis [268]. A placebo controlled RCT is underway with a planned inclusion of 900 patients given a dose of 900 mg for 6 months after surgery [269]. If this study confirms the results of previous studies, ursodeoxycholic acid should likely be recommended as prophylaxis against gallstone formation in patients without gallstones at the time of surgery. There is no data available on the potential effect of ursodeoxycholic acid to prevent progression of prevalent gall stones (Table 4). A systematic review of observational studies concluded that concomitant cholecystectomy for patients with symptomatic gallstones disease can be considered to be safe [270]. However, a sequential approach with a cholecystectomy before the bariatric surgery may be equally safe and efficient [271]. While the grade of evidence remains low, it is strongly recommended to consider cholecystectomy either before or at the time of bariatric surgery for patients with symptomatic gallstones disease.

#### Specific considerations in patients with diabetes

The consideration of diabetes is an addition to these guidelines [89].

In most reports of patients undergoing bariatric surgery, 15–20% of patients have T2D [200, 272, 273]. Carbohydrate loading is associated with an exaggerated hyperglycaemia in patients with diabetes. It is also recognized in several studies in patients undergoing non-bariatric surgery that hyperglycaemia is associated with worse outcomes, including increased complications and mortality in severe cases [274–276]. Therefore, the need to focus on the care of these patients is critical and the need for guidelines imminent.

In contrast to other types of surgery, bariatric surgery improves glucose homeostasis in patients with T2D, due to a variety of mechanisms, as early as in the immediate postoperative period [277]. Therefore, dose-adjustments in the glucose-lowering medications prescribed are often needed [278, 279]. This should be considered as early as possible since appropriate planning may facilitate early discharge and reduce LOS. Patients with diabetes who are prescribed preoperative LCD/VLCD should also be aware of the risk of hypoglycaemia during this period and might therefore be in need of adjustments of antidiabetic agents as well.

It should also be noted that some of the lessons learned from ERAS care in patients with diabetes undergoing bariatric surgery might be of relevance for other types of GI surgery, particularly oesophagogastric resections for cancer [280].

#### Final words

ERAS pathways include evidence-based items designed to reduce perioperative stress and maintain postoperative physiological function. The pathways were first adopted for colorectal surgery in 2005, but today ERAS guidelines are available for several fields of surgery [281]. Adherence to an updated ERAS protocol has been associated with reduced short- and long-term morbidity, as well as to improved recovery, shortened hospital LOS and reduced medical costs following major abdominal surgery [282, 283]. In modern bariatric surgery, the use of several items of the ERAS protocol has been widely accepted and shown to be associated with low rates of perioperative complications and faster recovery. However, the quality of evidence for many ERAS interventions is relatively low in a bariatric setting and evidence-based practices may need to be extrapolated from other surgeries. Higher quality of evidence would need additional confirmation from RCTs or large registries, and since some may often not be justified from an ethical perspective, or otherwise may not be feasible, the quality of evidence could be assumed to remain low. There is also a lack of studies addressing patient reported outcome measures as well as cost-effectiveness of some interventions. Although such studies may not improve quality of evidence in support of specific items, they may increase knowledge and provide a more complete understanding of the impact of specific interventions as well as clinical protocols.

Thus, the benefits of the adherence to an ERAS protocol in bariatric surgery have so far only been possible to demonstrate with high quality of evidence for functional recovery and hospital LOS [5, 6].

Since the first version of the ERAS guidelines for bariatric surgery was published in 2016 [7], results from new studies have impacted the level of evidence for certain recommendations, while others remain the same. The details of an ERAS pathway for patients with specific comorbidities who may have potentially more complex perioperative course, such as those with diabetes, cardiovascular and psychiatric comorbidities, deserve further focus. With the well documented effect of ERAS on reducing perioperative stress, the gains in terms of reduced perioperative morbidity could be expected to be particularly evident in these patient populations.
